# Cardiac magnetic resonance T2 relaxometry in acute myocarditis correlations with myocardial function and cardiac biomarkers

**DOI:** 10.1186/1532-429X-17-S1-P302

**Published:** 2015-02-03

**Authors:** Agnes Mayr, Pierre Schanen, Andreas Loewen, Michael Schocke

**Affiliations:** 1 Radiology, Medical University Innsbruck, Innsbruck, Austria

## Background

Myocarditis is a common inflammation of the myocardium with variable symptoms, which is in daily clinical routine quite difficult to diagnose. Cardiac magnetic resonance imaging assumes an increasingly important role in the diagnosis of myocarditis by their exact functional analysis and the possibility of representation of tissue changes. The aim of this clinical trial consists in the examination of the validity of cardiac biomarkers and cardiac function parameters in the context of magnetic resonance T2 signal intensity in myocarditis.

## Methods

This retrospective study includes the collection of data from 39 patients (n = 39) with a confirmed myocarditis by magnetic resonance imaging (MRI). In addition, next to the main criterion of radiologically diagnosed myocarditis, elevated cardiac biomarkers (troponin or creatine kinase), increased CRP or increased NT-proBNP were regarded as an inclusion criterion.

## Results

The maximum deflection of cardiac biomarkers showed a significant correlation with the T2 relaxation times in patients with myocarditis in the acute stage (CK: p> 0.034, r = 0.463; kTnT: p> 0.048, r = 0.384). The correlations between myocardial functional parameters and T2 relaxation times missed the statistical correlation level.

## Conclusions

In summary, the obtained results show that calculated T2 relaxation times in MRI are directly related to the biochemical severity of myocarditis. T2-mapping in MRI represents a stable method of detection of myocardial edema in myocarditis.

## Funding

Nothing to declare.

